# Conditional down-regulation of GreA impacts expression of rRNA and transcription factors, affecting *Mycobacterium smegmatis* survival

**DOI:** 10.1038/s41598-020-62703-7

**Published:** 2020-04-02

**Authors:** Rajiv Kumar Jha, Shubha Udupa, Ashutosh Kumar Rai, Phoolwanti Rani, Prakruti R. Singh, Shamitha Govind, Valakunja Nagaraja

**Affiliations:** 1 Department of Microbiology and Cell Biology, Indian Institute of Science, Bangalore, 560 012 India; 2 Jawaharlal Nehru Centre for Advanced Scientific Research, Bangalore, 560 064 India

**Keywords:** Microbiology, Molecular biology

## Abstract

Gre, one of the conserved transcription factors in bacteria, modulates RNA polymerase (RNAP) activity to ensure processivity and fidelity of RNA synthesis. Gre factors regulate transcription by inducing the intrinsic-endonucleolytic activity of RNAP, allowing the enzyme to resume transcription from the paused and arrested sites. While *Escherichia coli* and a number of eubacteria harbor GreA and GreB, genus mycobacteria has a single Gre (GreA). To address the importance of the GreA in growth, physiology and gene expression of *Mycobacterium smegmatis*, we have constructed a conditional knock-down strain of GreA. The GreA depleted strain exhibited slow growth, drastic changes in cell surface phenotype, cell death, and increased susceptibility to front-line anti-tubercular drugs. Transcripts and 2D-gel electrophoresis (2D-PAGE) analysis of the GreA conditional knock-down strain showed altered expression of the genes involved in transcription regulation. Among the genes analysed, expression of RNAP subunits (β, β’ and ω), *carD, hupB, lsr2, and nusA* were affected to a large extent. Severe reduction in the expression of genes of rRNA operon in the knock-down strain reveal a role for GreA in regulating the core components of the translation process.

## Introduction

Transcription is the central process in the cell. It is regulated by a variety of proteins at different stages. Many of these regulators control transcription by modulating the RNAP activity. The movement of RNAP along the template often gets interrupted by pauses resulting in backtracked RNAP. During backtracking, RNAP slides backwards along the DNA and the 3′ end of the newly synthesized RNA is disengaged from the active centre^1,2^. In order to continue RNA extension from the 3′ end, bacteria have evolved strategies that serve to assist the backtracked RNAP to reinitiate elongation. The newly synthesized 3′ end of RNA is subjected to intrinsic cleavage by RNAP itself^3–6^. Gre factors bind to the RNAP and assist transcript-cleavage^1,7–12^. The absence of Gre would prolong or even prevent rescuing of backtracked RNAP complexes leading to a pause or arrest of transcription. This would limit overall rate and processivity of transcription and hence cell survival.

Although Gre factors or their homologues are conserved in all forms of life, present understanding of their *in vivo* role in bacteria other than in *E. coli* is limited. Even in *E. coli*, a complete understanding of the effect of Gre mutants is hindered because of the presence of two Gre (GreA and GreB) and also the partial redundancy in function by the other secondary channel binding proteins such as DksA, Rnk and TraR^13–15^. While GreA cleaves 2–3 nucleotide from 3′ end of RNA, GreB cleaves up to 9 nucleotide fragments. However, how the other secondary channel binding proteins complement their function is not clearly understood. It has been shown that GreA, GreB and DksA are mutually competing and exhibit functional redundancy^15^. In *E. coli*, a double knock-out of *greA* and *greB* did not show lethality, although colonies were small and also hypersensitive to metal ions^16^. Deletion of GreA led to a 100-fold increase in transcription error compared to the wild-type (WT) *E. coli*. However, overexpression of GreB was able to rescue the increased misincorporation to some extent in *greA* knock-out strain revealing the backup function^17^.

Unlike *E. coli* and many other bacteria, the genomes of both *Mycobacterium tuberculosis* (*Mtb*) and *M. smegmatis* harbour a single *gre* factor with properties somewhat similar to GreA of *E. coli*. The factor cleaves only a few nucleotide from 3′ end of RNA and it shown to overcome abortive initiation^18^. Given the GC-richness of mycobacterial genomes, the GreA is likely to have greater impact during transcription. GC rich sequences can impose blockage during transcription due to the formation of more stable RNA-DNA hybrids and R-loops^19,20^. The transcription in mycobacteria may encounter such blockage and halt RNAP progression. The enzyme has to overcome this impediment and ensure completing the elongation process. Presence of a sole representative, lack of some of the other secondary channel binding proteins (DksA, TraR) would likely render the GreA function essential in mycobacteria. However, earlier saturation transposon mutagenesis studies suggested *Mtb*GreA to be a non-essential gene^21^. Examination of the transposon insertion site indicated that the insertion is at 493^rd^ nucleotide i.e. towards the C-terminal end of the gene. A much more sensitive high-density mutagenesis and deep-sequencing predicted its essentiality for *in vitro* growth of *Mtb*^22^. Overexpression of antisense RNA of *greA* in mycobacteria also suggested that it could be essential for mycobacterial growth^18^. However, the consequences of decreased level of GreA in the mycobacterial physiology is yet to be explored. Thus, to examine the importance of GreA in mycobacteria, a conditional knock-down of GreA in *M. smegmatis* was generated using CRISPRi^23^ and its requirement for growth and physiology was elucidated. The conditional knock-down with reduced expression of GreA exhibited growth defect, phenotypic variations, altered protein expression, and cell death indicating the essential role of GreA in maintaining the physiology of *M. smegmatis*.

## Results

### GreA is required for growth and cell survival

**Figure 1:**
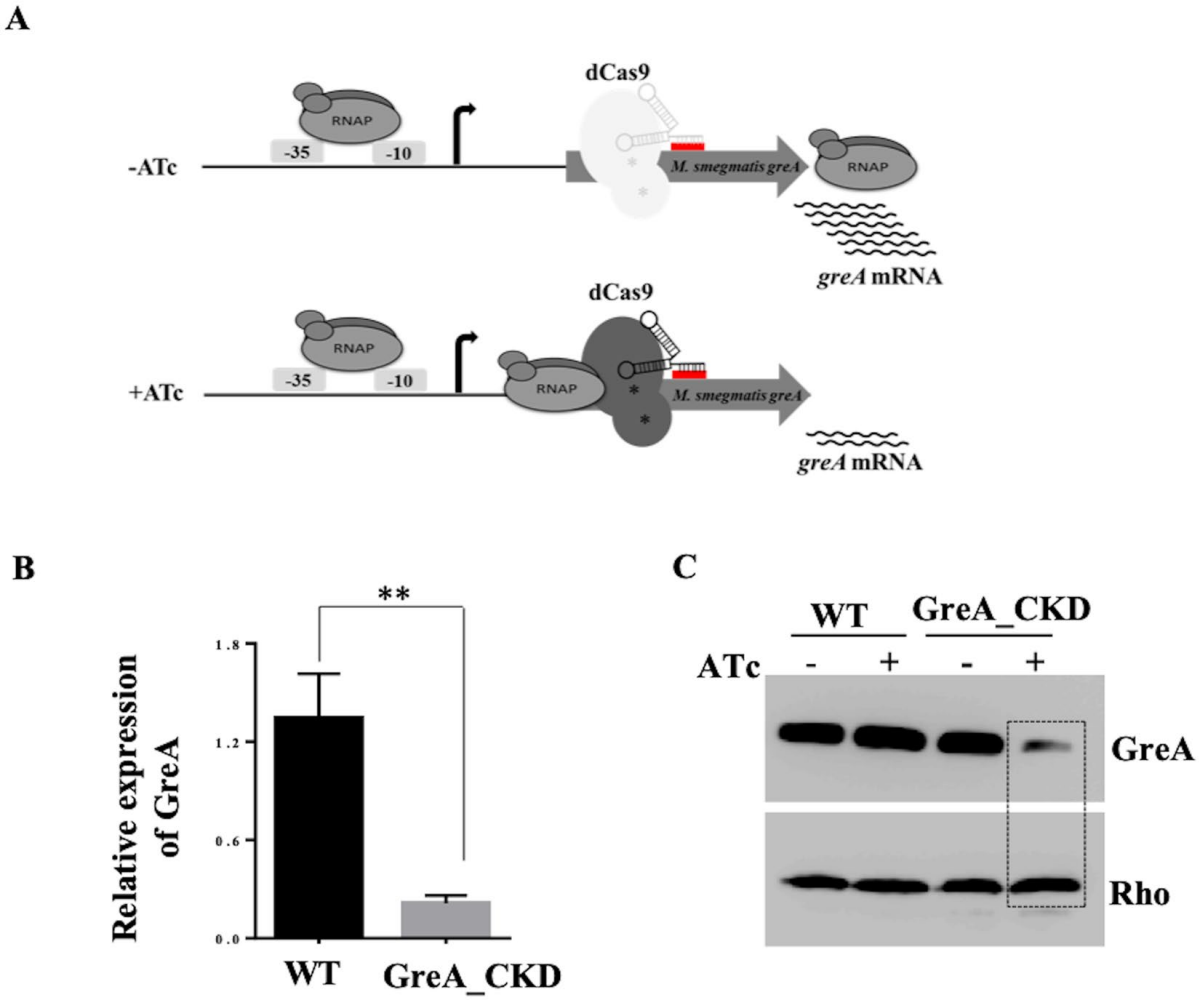
Construction of GreA conditional knock-down in *M. smegmatis*. (**A**) Schematic representing the strategy employed to generate a conditional GreA knock-down. In the presence of ATc, dCas9 along with a guide-RNA target the non-template strand of *greA* at +333 position and would lead to a transcription interference (**B**) The qRT-PCR data showing relative mRNA level of *greA* in the WT and GreA_CKD cells treated with ATc as compared to untreated cells at 30 °C. The level of 16 S rRNA in the knock-down strain was found to be reduced (see results), mRNA of another abundant protein Rho in mycobacteria was used for normalization of qPCR. (**C**) Immuno-blot showing the effect of ATc on GreA (upper panel) and Rho (lower panel) in GreA_CKD strain at 30 °C. The experiments were carried out three times and representative blots are shown. The full-length blots are presented in Supplementary Figure S2. GraphPad prism software was used for paired t-test statistical analysis. P-value < 0.05 was considered as significant. * ≤ 0.05, ** ≤ 0.01.

To understand the role of GreA and its requirement in *M. smegmatis*, a well-established CRISPRi technology^23^ was used to generate conditional knock-down in mycobacteria. In this approach, downregulation of the gene is effected by the roadblock for RNAP generated within the transcribed region. In the presence of ATc, the guide-RNA expressed would target non-template strand of *greA*, recruiting dCas9 to the site. The complex would impede the translocation of RNAP during transcription and thus attenuate *greA* expression (Fig. 1A). We used guide-RNA to target various regions of greA (data not shown). The guide-RNA at position +333 showed maximum level of GreA repression. The level of GreA in the strain was assessed by qRT-PCR and immunoblotting. Initially, we used 16 S rRNA as internal control to monitor the expression. However, while quantifying the *greA* transcript by qPCR, we observed severe reduction in Ct value of 16 S rRNA (see later section). Thus, for normalization of qPCR, we used *rho* mRNA which is abundant in mycobacteria and the protein level did not change during various mycobacterial growth phases and stress (Anirban Mitra, PhD Thesis 2013, Indian Institute of Science, Bangalore). Upon addition of ATc, the expression of *greA* in conditional knock-down strain was (GreA_CKD) reduced both at mRNA and protein level by 70–80% than the WT strain, whereas *rho* expression was unaffected (Fig. 1B,C).

**Figure 2:**
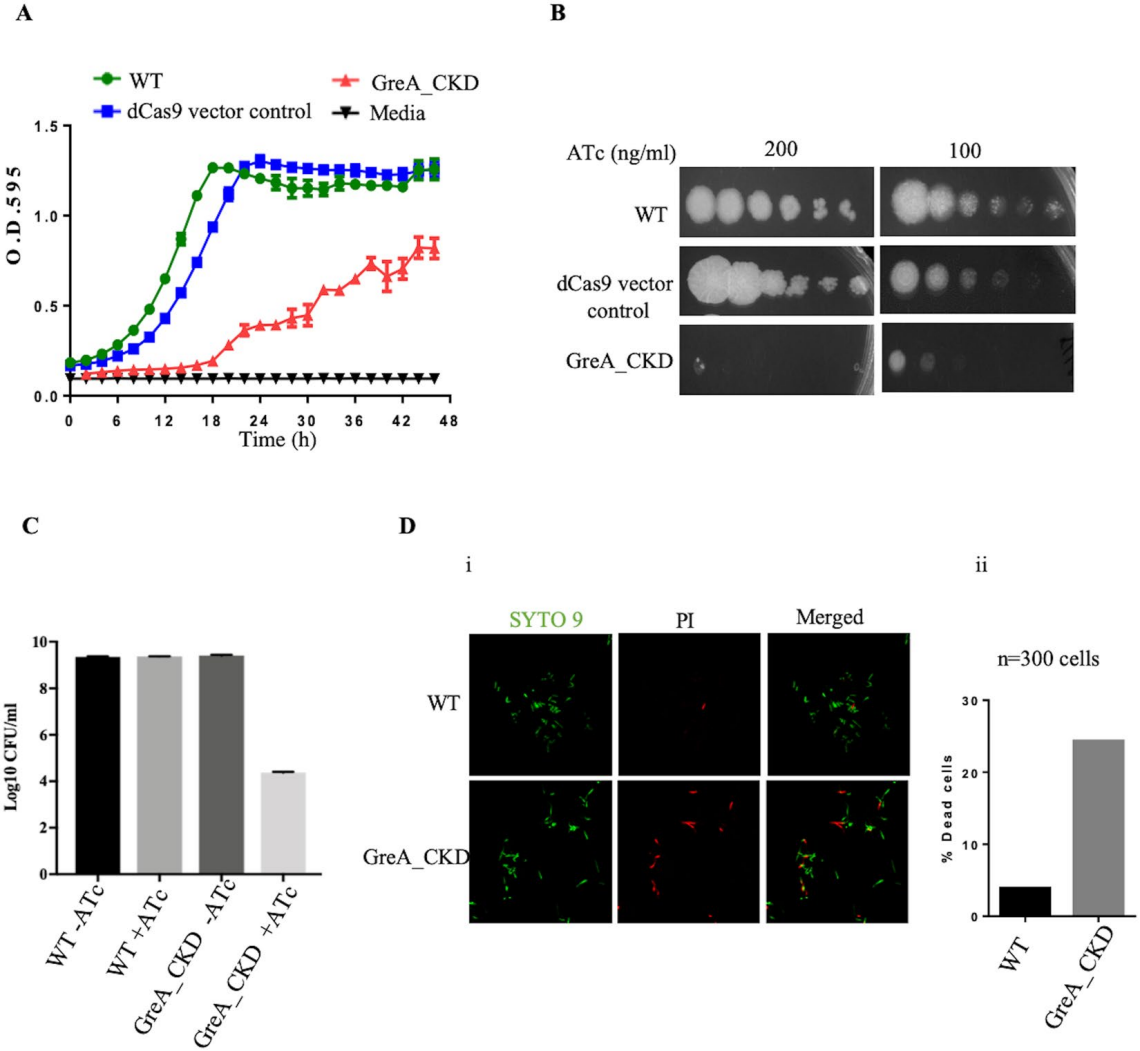
Reduction in GreA level affect growth and survival of *M. smegmatis*. (**A**) WT, dCas9 expressing vector, and GreA_CKD cultures were grown in Middlebrook 7H9 medium with ATc (200 ng/ml) and incubated at 30 °C. The growth was monitored by measurement of O.D._595_ every 2 h. The data was plotted using GraphPad prism software and error bar representing the SD from three biological replicates. (**B**) Exponential phase cultures were 10-fold serially diluted and spotted on Middlebrook 7H11 agar media supplemented with ATc. The plates were incubated at 30 °C and bacterial growth was monitored after 3-days of incubation. Experiments were carried out thrice with similar results. Representative images are shown. (**C**) WT and GreA_CKD cultures were grown till exponential phase and CFU analysis was carried out by plating on Middlebrook 7H11 agar media in the absence and presence of ATc (100 ng/ml). (**D**) WT and GreA_CKD cultures were grown till exponential phase in the presence ATc (200 ng/ml) and stained with Syto9 and propidium iodide (PI). Confocal microscopy images of WT and GreA_CKD cells stained with Syto9 are shown in green color (live cells) and with PI are indicated in red color (dead cells). In the right panel, the number of PI positive cells/300 bacteria from various fields was determined by confocal microscopy and plotted as percentage of dead cells.

To monitor the effect of GreA depletion on growth of *M. smegmatis*, the cultures were grown in 7H9-broth in the presence of ATc. The GreA depleted strain exhibited slow growth compared to both WT and dCas9 expressing vector control (Fig. 2A). To evaluate the effect of GreA down-regulation on the bacterial growth on the solid agar, the exponentially grown cultures of WT, GreA_CKD, and dCas9 expressing vector control were spotted on Middlebrook 7H11-agar plates. The GreA knock-down strain exhibited extreme slow growth (Fig. 2B) which was partially rescued when ATc concentration was reduced to 100 ng/ml instead of 200 ng/ml (Fig. 2B).

To examine whether GreA depletion impacted cell viability, CFUs were determined after the induction of the guide RNA and dCas9 to generate roadblock (Materials and Methods). From the data presented in Fig. 2C, it is apparent that GreA knock-down strain showed reduced survival. Further, the cells were stained with Syto9 (green) and PI (red) to score for live and dead cells respectively. In the microscopic analysis, about 24% of cells in the GreA_CKD strain appear to be dead (red) as compared to 3.9% in WT (Fig. 2D), indicating that GreA is required for bacterial survival.

The compromised growth of the knock-down strain could affect cell surface characteristics and cell morphology. Mycobacterial cells contain high lipid content in their cell wall which causes them to aggregate. When grown in a detergent-free media under static conditions, they form a solid surface associated biofilm at the bottom of the container or a floating pellicle at the air-liquid interface^24^. The conditional GreA_CKD strain showed reduced biofilm and pellicle formation (Figure S1A). To quantify the biofilm formation by WT and GreA_CKD, cultures were grown in polystyrene plates. Biofilm forming ability of the GreA_CKD was compromised as compared with the WT (Figure S1B). The GreA_CKD showed reduced sliding motility and did not form the typical halo like structure (Figure S1A). The GreA_CKD strain did not show significant difference in the nucleoid status when the cells were stained with DAPI and observed under fluorescence microscope. However, the cells appeared to be elongated when GreA level was reduced (Figure S1C). Reduced level of GreA thus confers pleotropic effects resulting in altered cell surface properties in the GreA_CKD strain of *M. smegmatis*.

### Increased susceptibility to drugs

**Figure 3:**
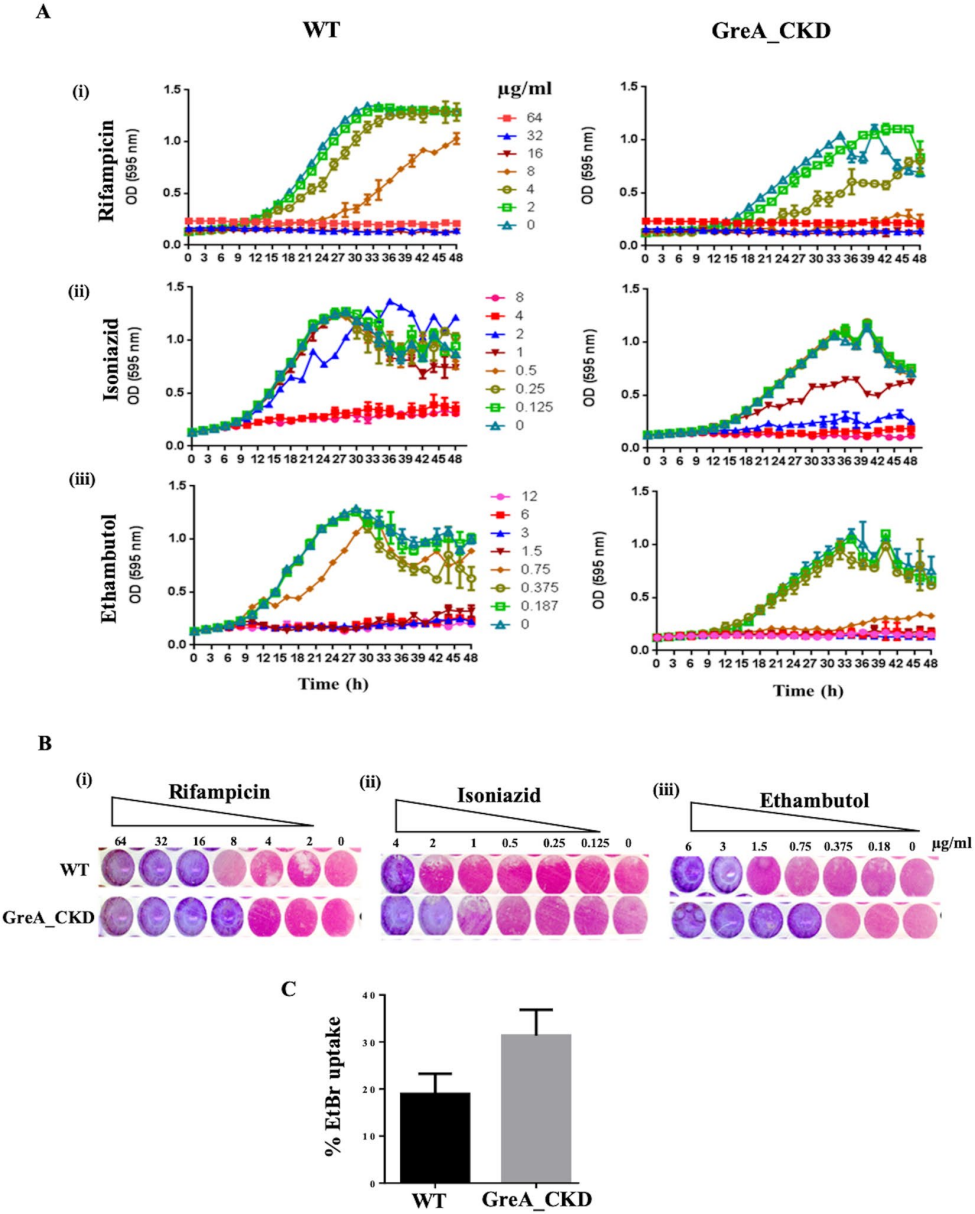
GreA_CKD strain showed increased susceptibility to the drugs. (**A**) Growth profile of WT and GreA_CKD strains in the presence of different concentrations of (i) rifampicin, (ii) isoniazid, and (iii) ethambutol. (**B**) Resazurin based plate assay showing the conversion of dye colour from pink (live cells) to blue (metabolic inactive cells) of the cultures grown in the presence of different concentration of (i) rifampicin, (ii) isoniazid, and (iii) ethambutol. (**C**) Histogram showing the percentage of EtBr uptake by WT and GreA_CKD cells. The results were plotted using GraphPad prism software and error bar representing the SD from three biological repeats.

Alteration in cell surface properties in the CKD strain could lead to alteration in cell permeability, resulting in increased susceptibility to antibiotics. The susceptibility of the bacteria to the drugs was assessed by growth and resazurin assay as described in Materials and Methods^25^. The GreA_CKD strain showed increased susceptibility to front-line anti tubercular drugs- rifampicin, ethambutol and isoniazid in comparison to the WT (Fig. 3A,B). In all the cases, at least two-fold higher sensitivity is seen in the CKD cells. For example, 4–8 ug/ml concentrations of rifampicin led to the cessation and delay in the growth of GreA depleted strain, whereas growth of WT strain was inhibited only at 16ug/ml (Fig. 3Ai,Bi,). While 1–2 ug/ml of INH (Isoniazid) would cause the growth arrest in GreA_CKD, the growth of WT strain was inhibited at 4 ug/ml (Fig. 3Aii,Bii). In the case of ethambutol, 0.75 ug/ml of drug treatment brought the growth inhibition of the CKD strain compared to the two- fold higher concentration required for inhibition of the WT strain (Fig. 3Aiii,Biii).

To test whether increased susceptibility to these drugs is due to the altered permeability of the cell upon reduction in GreA level, permeability was monitored by analysing the EtBr uptake by the GreA_CKD cells (Materials and Methods^26^,). The GreA_CKD exhibited more uptake of EtBr, indicating increased cell permeability compared to the WT (Fig. 3C).

### Depletion of Gre causes changes in the proteomic profile of *M. smegmatis*

**Figure 4:**
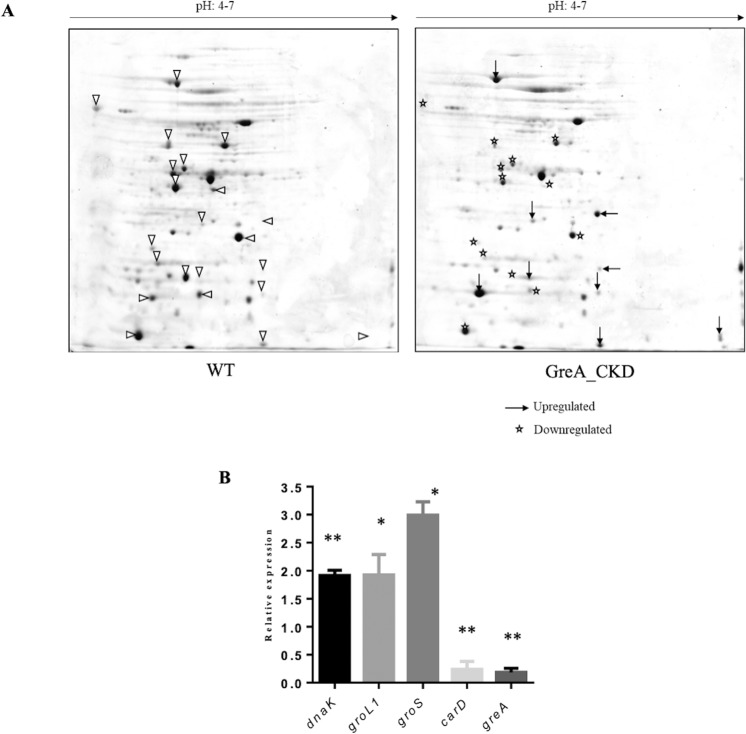
GreA deprivation affects the proteome. (**A**) Representative 2D-SDS gel showing the proteome profile of WT and GreA_CKD. Differentially expressed protein spots in GreA_CKD are highlighted with black arrow (upregulated) and asterisk (down regulated). **(B)** Differentially expressed proteins spots were subjected to MALDI-TOF analysis and validated with qRT-PCR analysis. The experiment was carried out three times and error bar is represents SD. GraphPad prism software was used for paired t-test statistical analysis. P-value < 0.05 was considered as significant. * ≤ 0.05, ** ≤ 0.01.

2D-gel electrophoresis was carried out to analyse the proteome of the GreA depleted strain. Comparison of the proteome profile of WT and GreA_CKD cells showed the differential expression of the proteins between the two strains (Fig. 4A). Among the clearly distinguishable differentially expressed proteins in GreA_CKD strain, 9 and 13 protein spots showed increased and decreased levels of expression respectively. The proteins were identified by peptide mass fingerprinting-mass spectroscopy. Transcription regulator CarD^27,28^, molecular chaperones GroL1, GroS and DnaK^29–34^, among the others with altered expression were chosen for further analysis. The changes in the expression of these proteins were confirmed by transcript analysis (Fig. 4B). Altered expression of many proteins in the CKD strain indicates the importance of GreA to maintain the regulated gene expression in *M. smegmatis*. These results correlate with the earlier observation in *Streptococcus pneumoniae* where knock-out of GreA affected the global gene expression^35^. Changes in phenotypes and proteome profile of GreA depleted *M. smegmatis* strain may impact its growth in the host. To investigate the growth of *M. smegmatis*, macrophages were infected with WT and GreA_CKD cells and bacterial survival was monitored after 24 h of infection by CFU analysis. No considerable changes in survival of GreA_CKD strain was observed till 24 h of infection (data not shown). Both WT and GreA_CKD cells were cleared out of the macrophages within 24 h infection, indicating that reduced GreA level may not be a critical determinant for *M. smegmatis* intracellular survival.

### Reduction in GreA alters expression of genes involved in transcription regulation

**Figure 5:**
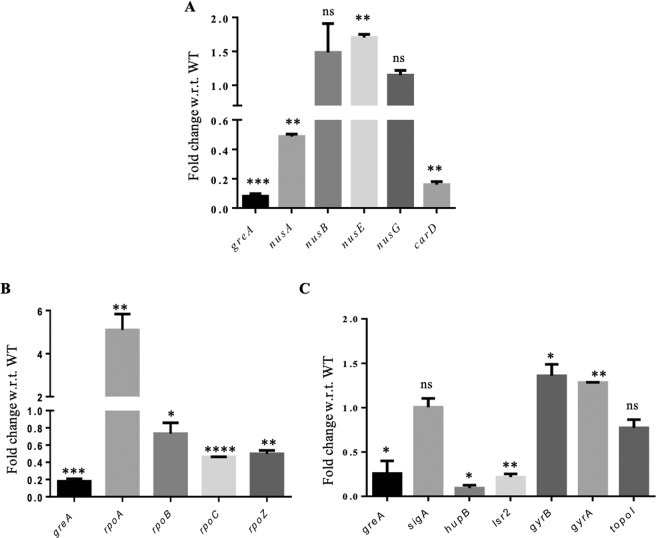
GreA deprivation affects expression of genes involved in transcription regulation. (**A**) Histogram showing the fold-change in the mRNA expression of RNAP-interacting proteins in the GreA_CKD compared to WT. **(B)** Histogram showing the expression of RNAP subunits in the GreA_CKD cells. **(C)** Expression profile of topology-modulators in the GreA_CKD compared to WT *M. smegmatis*. *rho* mRNA was used for normalization. The error bar represents the SD from three independent experiments. GraphPad prism software was used for paired t-test statistical analysis. P-value < 0.05 was considered as significant. ns- nonsignificant, * ≤ 0.05, ** ≤ 0.01, *** ≤ 0.001, **** ≤ 0.0001.

GreA is required for the proper transcription elongation, and perturbation in its level could alter the expression of other transcription regulators. Thus, it was no surprise to see the reduction in CarD^27^ in the proteome analysis. However, given the severity of GreA_CKD phenotype, one would have expected the impact on the expression of other factors that influence the transcription. To determine the effect of GreA depletion on other transcription regulators which interact with RNAP in *M. smegmatis*, transcript analysis of NusA, NusB, NusE, and NusG was carried out. CarD was used as a control (see Fig. 4B). NusE expression is marginally up-regulated, whereas NusA was down-regulated similar to CarD (Fig. 5A). However, the expression of NusG and NusB were unaffected by Gre depletion. Further, the expression of different subunits of RNAP (*rpoA, rpoB, rpoC* and *rpoZ*) was also altered in the GreA_CKD cells (Fig. 5B). Notably *rpoA* RNA expression was increased by 5–6-fold while the expression of other components of the enzyme was decreased. Next, the expression of genes encoding DNA-binding proteins *viz. topoI*, *gyrA*, *gyrB*, *hupB*, and *lsr2* which influence the topology homeostasis and transcription were monitored. The house keeping sigma, SigA served as control. The expression of two NAPs, HU and Lsr2 was significantly reduced in the GreA_CKD as compared to WT (Fig. 5C) suggesting that optimum levels of GreA is required to maintain the optimum level of these DNA binding proteins in *M. smegmatis*. The altered expression of these key transcription and topology modulators could be contributing to the observed changes in the phenotypes of GreA_CKD strain (see Discussion).

### Expression of rRNA-operon is dependent on GreA level

**Figure 6:**
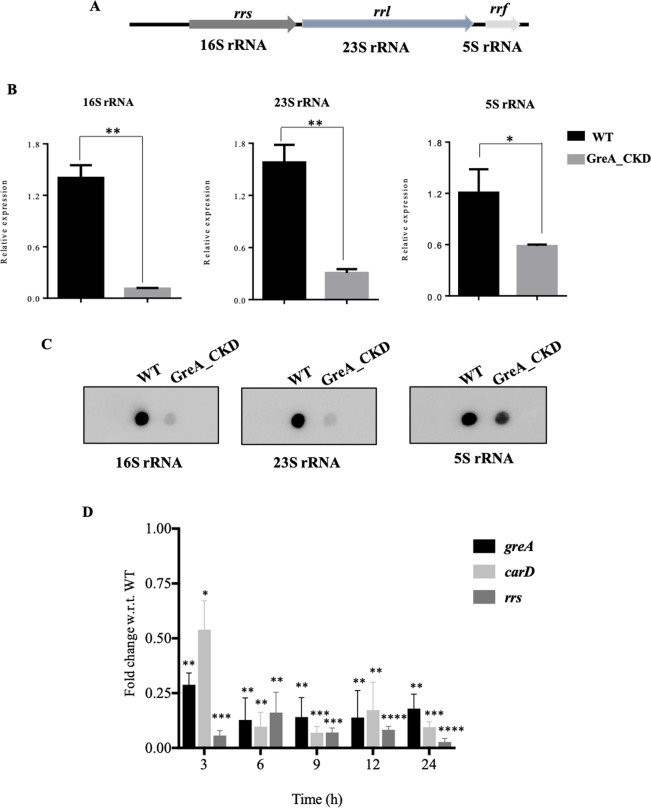
GreA is essential for expression of rRNA operon. (**A**) Organization of rRNA operon in *M. smegmatis*. **(B)** qRT-PCR data showing the expression of rRNA operon genes, (i) 16 S rRNA, (ii) 23 S rRNA, and (iii) 5 S rRNA. *rho* mRNA level was used for normalization. The experiments were repeated in three biological replicates. **(C)** Slot-blot showing the expression of (i) 16 S rRNA, (ii) 23 S rRNA, and (iii) 5 S rRNA genes in the WT and GreA_CKD strains. The experiments were carried out in duplicates and representative images are shown. **(D)** qRT-PCR data showing the expression of *greA*, *carD* and *rrs* in GreA_CKD strain with respect to WT at different time-points after ATc treatment. The error bar represents the SD from three independent experiments. Paired t-test statistical analysis was performed using GraphPad prism software. P-value < 0.05 was considered as significant. * ≤ 0.05, ** ≤ 0.01, *** ≤ 0.001, **** ≤ 0.0001.

During transcript analysis of various transcription regulators in GreA depleted cell, as mentioned earlier initially 16 S rRNA was used for normalization as it is the most preferred yardstick for accessing the relative levels of other transcripts. However, surprisingly, the level of 16 S rRNA was several fold lower in the GreA_CKD strain and hence was found to be not suitable for normalization. The transcript level of rRNA operon – 16 S rRNA, 23 S rRNA, and 5 S rRNA (Fig. 6A), were monitored in the GreA depleted cells. The expression analysis showed that the level of all the three genes in the operon were down-regulated in the GreA depleted strain in comparison to WT (Fig. 6B). The reduction in expression was ranging from 6–8 fold for 23 S and 16 S RNA respectively. Northern dot-blots using gene specific probes for 16 S rRNA, 23 S rRNA, and 5 S rRNA confirmed the drastic reduction in all three stable RNAs (Fig. 6C). Such a severe reduction in rRNA operon expression would impact protein synthesis leading to downstream deleterious consequences seen in GreA depleted cell. Previous studies showed that the expression of rRNA in mycobacteria is positively regulated by CarD^28,36^. In GreA_CKD, apart from the reduction in rRNA expression, the expression of CarD was also reduced (Earlier section, Figs. 4B and 5A). Thus the effect of Gre on rRNA expression could be indirect i.e. through CarD. Hence, time-course qRT-PCR experiments were carried out with WT and GreA_CKD to determine whether the effect on rRNA expression is direct or through CarD. The analysis showed that the level of both CarD and 16 S rRNA were significantly reduced within 3 hours of ATc treatment (Fig. 6D), suggesting the direct role of GreA in regulating both CarD and 16 S rRNA. The decrease in expression of 16 S rRNA appears to be due to the combined effect of reduction in both CarD and GreA. Taking all the data from Figs. 4 to 6 together, it is apparent that the reduction in Gre level severely impacts the expression of several housekeeping functions *viz*, RNAP, rRNA, transcription regulators, NAPs, -eventually impacting growth and viability of the bacterium.

## Discussion

We describe the effect of reduction in the level of a single transcript cleavage factor GreA in *M. smegmatis*. The factor is required for normal growth and survival of the organism. Knocking down of *greA* resulted in altered expression of various topology and transcription regulators. The expression of rRNA operon genes is positively regulated by GreA. Depletion of GreA level in *M. smegmatis* influenced the proteome of the cell and altered cell surface phenotype.

During transcription elongation, backtracking of RNAP is seen especially at transcript pause sites^1,11,12,18^. RNAP pausing and backtracking could also occur during late stages of transcription initiation^37–39^. The dedicated function of Gre factors is to act on backtracked RNAP ternary complexes. When the level of Gre factors reduced, such backtracked complexes would accumulate in large excess leading to the inactivation of the RNAP complexes. Although the genome of *M. smegmatis* is larger than *E.coli* (6.7 Mb vs. 4.6), RNAP secondary channel binding proteins are underrepresented. *M. smegmatis* has a Gre homolog (encoded by MS-6292), but our previous studies revealed that it does not function like a Gre protein^40^. Thus, unlike *E.coli* and other eubacteria which have the luxury of having additional secondary channel binding proteins to ‘stand in’ for Gre function, no such functional redundancy is seen in *M. smegmatis*, leading to drastic consequences in GreA_CKD strain. It is a moot point why mycobacteria has a single Gre while *E.coli* and many other bacteria have additional factors.

As GreA is involved in rescuing the paused or stalled RNAPs during transcription elongation, reduction in its level would affect the movement of RNAP on the DNA template, impacting the expression of a number of genes. In *B. subtilis*, inactivation of GreA led to the accumulation of RNAPs at many promoters and promoter-proximal regions of highly expressed genes. However, no changes were seen in gene expression and phenotype^41^ possibly due to the functional input from other factors as in the case of *E.coli*. In contrast, GreA from *S. pneumoniae* appears to be required for growth and gene expression^35^. The organism has a single Gre factor as in the case of mycobacteria. However, the GreA deletion cells survive with increased doubling time and the expression of highly transcribed genes were reduced. By stochastic simulation analysis, a model was proposed in which queing of the trailing RNAPs at unresolved backtracked complexes could account for transcription traffic jams in the absence of GreA^35^. Notably, in contrast to *M.smegmatis*, no significant changes in the rRNA operon expression was observed. In *M. smegmatis*, the importance of GreA is realized by the fact that the GreA_CKD strain showed altered expression of many topology and transcription regulators; expression of genes encoding of RNAP subunits, *rpoB*, *rpoC and rpoZ* were also reduced. Moreover, expression of highly transcribed genes of rRNA operon were found to be affected (Fig. 6). The rRNA operon genes 16 S rRNA, 23 S rRNA, and 5 S rRNA are the integral components of protein syntheis and decrease in their level would jeopardise the overall translation process. The optimal expression of these essential components is required to maintain normal growth and metabolic activities of a cell. Perturbation in the expression of all these genes would in turn severely affect the expression of many other genes directly or indirectly. The changes in the phenotype and expression of proteins in the GreA depleted strain thus could be the consequence of the altered gene expression of all these key players. While the down regulation seen can be explained as a direct consequence of GreA depletion, upregulation of a few genes (*rpoA*, *nusE*) seen is likely to be an indirect effect. The expression of regulatory proteins which influence the expression of these genes are likely to be impacted upon GreA depletion leading to their increased expression.

One of the outcomes of this study is the connection between GreA and CarD in *M. smegmatis* in regulating rRNA operon. Among all the factors tested for alteration in the expression level in the GreA_CKD, CarD expression was most severely affected. CarD is an essential transcription factor in both *M. smegmatis and M. tuberculosis*^27^. Notably it is absent in *E.coli*. By binding to RNAP and DNA promoter elements it regulates transcription initiation^28^. Importantly it is involved in stabilizing RNAP-promoter open complexes which are otherwise unstable in mycobacteria^40,42,43^. Its essentiality also stems from its concentration dependent transcription activation at different promoters^42^. Thus decrease in CarD levels would impact transcription initiation at several genes. When CarD was discovered, it was found to be a negative regulator as its depletion led to upregulation of 16 S rRNA^27^. However, in a subsequent study, it was shown to be a global regulator of transcription initiation^28,36^. Trancript analysis of CarD depleated *M.smegmatis* showed differential regulation of large number of genes including rRNA and translational machinary genes^27^. CarD specifically activated transcription from rRNA promoters in *M.tuberculosis*^28^. The activation mechanism involved stabilization of open complex formation by increasing the isomerization rate and decreasing the rate of DNA closing^42,44^. The decrease in CarD expression upon GreA depletion thus appears to adversely affect the rRNA transcription.

Several explanations would account for drastic reduction of rRNA genes expression in the GreA_CKD cells. The depletion of GreA level would impact the transcription initiation as well as elongation as backtracked RNAP complexes would accumulate. Our prior promoter: RNAP interaction studies revealed the distinct rate limiting steps of mycobacterial promoters. Notably the rRNA promoters from both *M. smegmatis* and *M. tuberculosis* were unstable^43,45^. Among the two rRNA promoters, P_*rrnAPCL1*_ and P_*rrnB*_, the former showed a high level of abortive initiation^45^. The decrease in rRNA expression in part can be explained by the observation that GreA is also an activator of transcription by rescuing the abortive initiation^18^. Further, as described above, the reduction in CarD level in the GreA_CKD would also impact rRNA transcription initiation. Thus, GreA is a very crucial factor in controlling rRNA transcription by its direct role as well as through CarD. As both are essential for cell survival in mycobacteria, the impact of GreA knock-down is severely manifested.

## Materials and Methods

### Bacterial strains, plasmids and growth conditions

*M. smegmatis* and variants of the strain were cultured in Middlebrook 7H9 broth (Difco) or on 7H11 agar plates (Difco), supplemented with 0.2% glycerol and 0.05% Tween-80 at 30 °C. Antibiotics were added to the media at the following concentrations kanamycin, 25 μg/ml, and hygromycin, 50 μg/ml. For induction of sgRNA and dCas9 expression, cultures were supplemented with anhydrotetracycline (ATc) to achieve a final concentration of 100 ng/ml or 200 ng/ml. The strains of bacteria and plasmids used in this study are listed in Supplementary Table S1.

### Construction of conditional knock-down of GreA in *M. smegmatis*

The conditional knock-down strain was generated using CRISPRi based method as described^23^. In brief, guide-RNA targeting the non-template strand of *greA* at +333 position (Supplementary Table S2) was cloned into pRH2521 plasmid (hygromycin resistant) at BbsI site. The resulting construct, MsGreA_pRH2521 was transformed into *M. smegmatis* cells in which pRH2502 (for dCas9 expression, kanamycin resistant) had been previously introduced. The colonies were selected on the 7H11-agar plates containing hygromycin (50 µg/ml) and kanamycin (25 µg/ml). The expression of GreA in the conditional knock-down strain (GreA_CKD) was monitored by real-time qPCR and immunoblotting.

### Growth analysis of GreA_CKD

To monitor the growth in liquid medium, primary cultures of wild type, dCas9 expressing vector control, and GreA_CKD were grown to mid log phase (O.D. _595nM_ = 0.6–0.8) and diluted to O.D. _595nM_ = 0.02 with 7H9 medium. Cultures were grown for 3 h and then supplemented with anhydrotetracycline (ATc). The O.D. _595nM_ was measured every 3 h at 30 °C using the BioScreen growth curve analyzer, and plots were generated using GraphPad prism. To monitor the growth of wild type, dCas9 expressing vector control, and GreA_CKD cells on solid-agar medium, the cultures were spotted on 7H11-agar plate containing different concentration of ATc (0–200 ng/ml) and incubated at 30 °C.

To determine CFUs, WT and GreA_CKD cells were grown till O.D. _595nM_ 0.2 and treated with ATc (0–200 ng/ml). Aliquots of treated cultures (O.D. _595nM_ = 0.6) were serially diluted in 7H9 broth. 100 ul of the appropriate dilutions were spread on the 7H11 agar plates and incubated at 30 °C for 72 h before CFU were counted.

### RNA isolation and qRT-PCR

Cells (WT and GreA_CKD) grown with or without ATc, harvested, and RNA was isolated using RNAzol RT (Sigma) according to manufacture protocol. The RNA was treated with DNase I (Turbo) to remove the traces of chromosomal DNA and tested for DNA contamination using PCR. Equal amount of total RNA (quantified using Nanodrop spectrophotometer) from each strains were used for cDNAs synthesis (cDNA reverse transcription kit, Applied Biosystems). cDNA generated with random primers was used for qPCR, with SYBR green as the indicator dye. Expression of the genes was quantified after normalization of RNA levels to expression of the *rho* gene. Since the level of 16 S rRNA in the knock-down strain was found to be reduced (see results), mRNA of another abundant protein Rho in mycobacteria (Anirban Mitra, PhD Thesis 2013, Indian Institute of Science, Bangalore) was taken for normalization of qPCR data. The primers for RT-qPCR were designed to generate amplicons of 100–150 nucleotides (Supplementary Table S2).

### Immunoblot analysis

Equal amount cell lysates were separated on SDS PAGE (15%) and transferred to PVDF membranes. The membranes were incubated in blocking buffer [10 mM Na-phosphate, pH 7.5, 150 mM NaCl, 20 and 2% (W/V) BSA] for 2 h, and then with rabbit-raised anti-GreA^18^, and anti-Rho^46^ primary antibodies diluted (1:10000) in 1×-PBS with 2% BSA for 2 h followed by 10 min washing in PBST (1×-PBS with 0.05% Tween 20) for three times. The membrane was incubated with HRP-conjugated secondary antibody (1:20000) in PBS with 2% BSA (GE Amersham) for 2 h, and developed using chemiluminescent HRP-substrate (Millipore).

### Biofilm, pellicle and motility assays

WT and GreA_CKD cells were inoculated to an O.D._595_ of 0.02 in 7H9 medium without Tween 80 in a 12-well cell culture plates (Bio-fill). ATc was added to a final concentration of 200 ng/ml. The plates were incubated at 30 °C for 48 h without disturbance. Biofilms were quantified using crystal violet (CV) staining as described earlier^24,47^. Briefly, free-floating cells were washed with deionized water, stained with 1% crystal-violet, and assayed by spectrophotometric reading of the ethanol extract at 570 nm. Pellicle formation was monitored by growing the cultures without shaking at 30 °C in Middlebrook 7H9 medium devoid of Tween 80. Sliding motility assays were carried out as described previously^48^. Briefly, 2 µl of exponential phase cultures were spotted on motility medium consisting of 7H9 supplemented with 0.5% Casamino acids and 0.2% glycerol, solidified with low-melting-point agarose (0.4%, w/v). The plates were incubated for 24–96 h at 30 °C.

### Drug sensitivity assessment

Primary cultures of WT and GreA_CKD cells were diluted to an O.D._595_ = 0.02, treated with ATc (200 ng/ml) and grown for 3 h. The cultures were aliquoted into X100 Bioscreen sterile plates honeycomb (ThermoFisher), followed by addition of anti-TB drugs. Untreated cells were taken as control and growth monitored at O.D._595 with_ continuous shaking at 30 °C in the Bioscreen growth analyzer. To monitor cell viability, resazurin reduction microplate assay was carried out^25^. Resazurin dye was added to the cultures at a final concentration of 0.05%, and the cultures were incubated at 37 °C for 3 h.

### EtBr uptake

The exponentially grown ATc treated culture was diluted to O.D. _595nM_ = 0.2 with PBS, washed, resuspended in 1X PBS and treated with Ethidium Bromide (0.5 µg/ml). The 200 µl of EtBr-loaded cells were aliquoted to 96 well black plate (Thermo fisher). The absorption and emission were measured at 515 nm and 600 nm wavelengths respectively. The EtBr uptake by the WT and GreA_CKD cells were plotted as percentage considering uptake by heat killed cells as 100%.

### 2D-gel electrophoresis and MS (2D-MS)

2D-gel electrophoresis was performed using the conventional IEF/SDS-2D PAGE as described earlier^49^. In brief, cultures of both WT and GreA_CKD were grown in 7H9 medium to an O.D._595_ = 0.6 in the presence ATc. Cells were harvested, washed (with PBS), and resuspended in lysis buffer (40 mM Tris/HCl buffer, pH 8.0, containing 1 mM PMSF, and lysed by bead beating. The lysate was spun at 13000 RPM for 30 min at 4 °C and supernatant was collected. For normalization, the concentration of total proteins in the lysate was quantified by Bradford method^50^ and equal amount of soluble proteins (1.2 mg) from both WT and GreA_CKD were processed with a ReadyPrep 2D Clean-up kit (Bio-Rad). Samples were applied to immobilized pH gradient (IPG) strips with a linear separation range of 4–7 pH (Bio-Rad). IEF was carried out on a protean i12 IEF Cell (Bio-Rad) using the following protocol: (a) 250 V, 2 h; (b) 500 V constant for 2 h; (c) 1000 V, 1 h, (d) 5000 V, 2 h and (e) 8000 V constant until 65kVh. The current limit was set at 50 µA/strip. After equilibration, the strips were loaded and resolved on 12% SDS-PAGE gels. The gels were stained with a ProteoSilver kit (Sigma-Aldrich), and the spots which differed in intensity between samples were identified by in-gel trypsin digestion and MALDI-TOF/TOF mass-spectrometry (MS). The peptide mass list were analysed by MASCOT software against the NCBI database using a setting described previously^51^. In brief, a peptide mass tolerance of ±1.2 Da, MS/MS tolerance of ±0.6 D, carbamidomethylation (C) as a fixed modification and oxidation (M) as a variable modification were set. A probability score of P < = 0.05 was used as the criterion for identification.

### Fluorescence (DAPI) microscopy

WT and GreA_CKD cells were sub-cultured in the 7H9 medium containing 0.2% glycerol to O.D. 595 nM = 0.4 in the presence of 200 ng/ml of ATc, harvested, and washed twice with 1X PBS followed by overnight fixation (1% TritonX-100, 2% toluene prepared in 1X PBS) at 4 °C. Prior to staining of the DNA, cells were washed and resuspended in PBS buffer [10 mM Na-phosphate, pH 7.5, 150 mM NaCl, 2] followed by treatment with lysozyme (2 mg/ml) for permeabilization. The cells were stained with DAPI (0.5 μg/ml) for 10 min and visualized in a ZeissLSM-710 microscope under 100 X oil immersion objective.

### Bacterial survival assay in THP-1 cells

Approximately 20,000 THP-1 cells (PMA differentiated) were infected with either WT or GreA_CKD strain (OD_600_ of 0.6–0.8) in a 96-well plate at a MOI of 30 for 4 h at 37 °C as described earlier^52^. Cells were washed with pre-warmed RPMI and amikacin treatment (0.2 mg/ml for 2 h) was given to remove extracellular bacteria. Cells were again washed, fresh RPMI media was added and infected macrophages were incubated at 37 °C in 5% CO_2_ incubator. Following infection, macrophages were lysed using 0.06% SDS-7H9 medium and released bacteria were serially diluted and plated on OADC-7H11 agar medium to determine CFU.

### Northern blot analysis

To analyse the change in the expression level of rRNA *in vivo*, northern dot-blots were performed. Briefly, 2 µg of RNA from WT and GreA_CKD were spotted on Nytran (H + hybond) membrane, and RNA was fixed by UV crosslinking (1200 energy, CL 1000 ultraviolet crosslinker). The blots were probed with P^33^-labelled 16 S, 23 S and 5 S rRNA internal region probes (Supplementary Table S2) and images were developed using phosphor imager.

## Supplementary information

Supplementary information.

## References

[1] Nudler E, Mustaev A, Lukhtanov E, Goldfarb A (1997). The RNA-DNA hybrid maintains the register of transcription by preventing backtracking of RNA polymerase. Cell.

[2] Komissarova N, Kashlev M (1997). Transcriptional arrest: *Escherichia coli* RNA polymerase translocates backward, leaving the 3’ end of the RNA intact and extruded. Proc. Natl. Acad. Sci. USA.

[3] Orlova M, Newlands J, Das A, Goldfarb A, Borukhov S (1995). Intrinsic transcript cleavage activity of RNA polymerase. Proc. Natl. Acad. Sci. USA.

[4] Zenkin N, Yuzenkova Y, Severinov K (2006). Transcript-assisted transcriptional proofreading. Science.

[5] Yuzenkova Y, Zenkin N (2010). Central role of the RNA polymerase trigger loop in intrinsic RNA hydrolysis. Proc. Natl. Acad. Sci..

[6] Mishanina TV, Palo MZ, Nayak D, Mooney RA, Landick R (2017). Trigger loop of RNA polymerase is a positional, not acid-base, catalyst for both transcription and proofreading. Proc. Natl. Acad. Sci. USA.

[7] Roghanian M, Yuzenkova Y, Zenkin N (2011). Controlled interplay between trigger loop and Gre factor in the RNA polymerase active centre. Nucleic Acids Res..

[8] Opalka N (2003). Structure and function of the transcription elongation Ffactor GreB bound to bacterial RNA polymerase. Cell.

[9] Martinez-Rucobo FW, Cramer P (2013). Structural basis of transcription elongation. Biochim. Biophys. Acta - Gene Regul. Mech..

[10] Laptenko O, Lee J, Lomakin I, Borukhov S (2003). Transcript cleavage factors GreA and GreB act as transient catalytic components of RNA polymerase. Embo j..

[11] Borukhov S, Sagitov V, Goldfarb A (1993). Transcript cleavage factors from *E. coli*. Cell.

[12] Borukhov S, Polyakov A, Nikiforov V, Goldfarb A (1992). GreA protein: a transcription elongation factor from *Escherichia coli*. Proc. Natl Acad. Sci. USA.

[13] Rutherford ST (2007). Effects of DksA, GreA, and GreB on transcription initiation: insights into the mechanisms of factors that bind in the secondary channel of RNA polymerase. J. Mol. Biol..

[14] Blankschien MD (2009). TraR, a homolog of a RNAP secondary channel interactor, modulates transcription. PLoS Genet..

[15] Vinella D, Potrykus K, Murphy H, Cashel M (2012). Effects on growth by changes of the balance between GreA, GreB, and DksA suggest mutual competition and functional redundancy in *Escherichia coli*. J. Bacteriol..

[16] Susa M, Kubori T, Shimamoto N (2006). A pathway branching in transcription initiation in *Escherichia coli*. Mol. Microbiol..

[17] Bubunenko MG (2017). A Cre transcription fidelity reporter identifies GreA as a major RNA proofreading factor in *Escherichia coli*. Genetics.

[18] China A, Mishra S, Nagaraja V (2011). A Transcript cleavage factor of *Mycobacterium tuberculosis* important for its survival. PLoS One.

[19] Belotserkovskii BP (2010). Mechanisms and implications of transcription blockage by guanine-rich DNA sequences. Proc. Natl. Acad. Sci..

[20] Loomis EW, Sanz LA, Chédin F, Hagerman PJ (2014). Transcription-associated R-Loop formation across the human FMR1 CGG-repeat region. PLoS Genet..

[21] Sassetti CM, Boyd DH, Rubin EJ (2003). Genes required for mycobacterial growth defined by high density mutagenesis. Mol. Microbiol..

[22] Griffin JE (2011). High-resolution phenotypic profiling defines genes essential for mycobacterial growth and cholesterol catabolism. PLoS Pathog..

[23] Singh AK (2016). Investigating essential gene function in *Mycobacterium tuberculosis* using an efficient CRISPR interference system. Nucleic Acids Res..

[24] Ghosh S, Indi SS, Nagaraja V (2013). Regulation of lipid biosynthesis, sliding motility, and biofilm formation by a membrane-anchored nucleoid-associated protein of *Mycobacterium tuberculosis*. J. Bacteriol..

[25] Palomino, J.-C. *et al*. Resazurin microtiter assay plate: simple and inexpensive method for detection of drug resistance in *Mycobacterium tuberculosis*. *Antimicrob Agents Chemother* **46**, 2720–2722 (2002). 10.1128/AAC.46.8.2720-2722.2002 PMC127336 12121966

[26] Rodrigues L, Ramos J, Couto I, Amaral L, Viveiros M (2011). Ethidium bromide transport across *Mycobacterium smegmatis* cell-wall: correlation with antibiotic resistance. BMC Microbiol..

[27] Stallings CL (2009). CarD is an essential regulator of rRNA transcription required for *Mycobacterium tuberculosis* persistence. Cell.

[28] Srivastava DB (2013). Structure and function of CarD, an essential mycobacterial transcription factor. Proc. Natl. Acad. Sci. USA.

[29] Li K (2012). Transcription elongation factor GreA has functional chaperone activity. PLoS One.

[30] Fan M (2012). The unusual mycobacterial chaperonins: evidence for *in vivo* oligomerization and specialization of function. Mol. Microbiol..

[31] Colaco CA, MacDougall A (2014). Mycobacterial chaperonins: the tail wags the dog. FEMS Microbiol. Lett..

[32] Fujiwara K, Ishihama Y, Nakahigashi K, Soga T, Taguchi H (2010). A systematic survey of *in vivo* obligate chaperonin-dependent substrates. EMBO J..

[33] Calloni G (2012). DnaK functions as a central hub in the *E. coli* chaperone network. Cell Rep..

[34] Fay A, Glickman MS (2014). An essential nonredundant role for mycobacterial DnaK in native protein folding. PLoS Genet..

[35] Yuzenkova Y (2014). Control of transcription elongation by GreA determines rate of gene expression in *Streptococcus pneumoniae*. Nucleic Acids Res..

[36] Zhu DX, Garner AL, Galburt EA, Stallings CL (2019). CarD contributes to diverse gene expression outcomes throughout the genome of *Mycobacterium tuberculosis*. Proc. Natl. Acad. Sci. USA.

[37] Duchi D (2016). RNA polymerase pausing during initial transcription. Mol. Cell.

[38] Lerner E (2016). Backtracked and paused transcription initiation intermediate of *Escherichia coli* RNA polymerase. Proc. Natl. Acad. Sci..

[39] Dulin D (2018). Pausing controls branching between productive and non-productive pathways during initial transcription in bacteria. Nat. Commun..

[40] China A, Mishra S, Tare P, Nagaraja V (2012). Inhibition of *Mycobacterium tuberculosis* RNA polymerase by binding of a Gre factor homolog to the secondary channel. J. Bacteriol..

[41] Kusuya Y, Kurokawa K, Ishikawa S, Ogasawara N, Oshima T (2011). Transcription factor GreA contributes to resolving promoter-proximal pausing of RNA polymerase in *Bacillus subtilis* cells. J. Bacteriol..

[42] Rammohan J, Ruiz Manzano A, Garner AL, Stallings CL, Galburt EA (2015). CarD stabilizes mycobacterial open complexes via a two-tiered kinetic mechanism. Nucleic Acids Res..

[43] China A, Tare P, Nagaraja V (2010). Comparison of promoter-specific events during transcription initiation in mycobacteria. Microbiology.

[44] Jensen, D., Manzano, A. R., Rammohan, J., Stallings, C. L. & Galburt, E. A. CarD and RbpA modify the kinetics of initial transcription and slow promoter escape of the *Mycobacterium tuberculosis* RNA polymerase. *Nucleic Acids Res*. **47**, 6685-6698 (2019). 10.1093/nar/gkz449 PMC6648326 31127308

[45] Tare P, China A, Nagaraja V (2012). Distinct and contrasting transcription initiation patterns at *Mycobacterium tuberculosis* promoters. PLoS One.

[46] Mitra A, Misquitta R, Nagaraja V (2014). *Mycobacterium tuberculosis* Rho is an NTPase with distinct kinetic properties and a novel RNA-binding subdomain. PLoS One.

[47] Chen JM (2006). Roles of Lsr2 in colony morphology and biofilm formation of *Mycobacterium smegmatis*. J. Bacteriol..

[48] Martínez A, Torello S, Kolter R (1999). Sliding motility in mycobacteria. J. Bacteriol..

[49] O’farrells PH (1975). High resolution two-dimensional electrophoresis of proteins. J. Biol. Chem..

[50] Bradford M (1976). A rapid and sensitive method for the quantitation of microgram quantities of protein utilizing the principle of protein-dye binding. Anal. Biochem..

[51] Perkins, D. N., Pappin, D. J. C., Creasy, D. M. & Cottrell, J. S. Probability-based protein identification by searching sequence databases using mass spectrometry data. *Electrophoresis* **20**, 3551–3567 (1999). 10.1002/(SICI)1522-2683(19991201)20:18<3551::AID-ELPS3551>3.0.CO;2-2 10612281

[52] Falcone V, Bassey E, Jacobs W, Collins F (1995). The immunogenicity of recombinant *Mycobacterium smegmatis* bearing BCG genes. Microbiology.

