# Supplementary material for: Conditional down-regulation of GreA impacts expression of rRNA and transcription factors, affecting Mycobacterium smegmatis survival
Source: Sci Rep. 2020 Apr 2;10:5802. doi: 10.1038/s41598-020-62703-7 (PMC7118132; doi:10.1038/s41598-020-62703-7)
Supplement: Supplementary file 1 — Supplementary information. [file 41598_2020_62703_MOESM1_ESM.pdf]

Conditional down-regulation of GreA impacts expression of rRNA and transcription factors, affecting *Mycobacterium smegmatis* survival

Rajiv Kumar Jha[1#], Shubha Udupa[1#], Ashutosh Kumar Rai[1], Phoolwanti Rani[1], Prakruti R Singh[1], Shamitha Govind[1], and Nagaraja V[1, 2]*

[1]Department of Microbiology and Cell Biology, Indian Institute of Science, Bangalore 560 012, India.

[2]Jawaharlal Nehru Centre for Advanced Scientific Research, Bangalore 560 064, India.

#These authors contributed equally to this research.

*Correspondence: Prof. Valakunja Nagaraja, [1]Department of Microbiology and Cell Biology, Indian Institute of Science, C.V. Raman Avenue, Bangalore 560 012, India; [2]Jawaharlal Nehru Centre for Advanced Scientific Research, Bangalore 560 064, India.
Email: vraj@iisc.ac.in, Tel. (91) 80 23600668; Fax. (91) 80 23602697

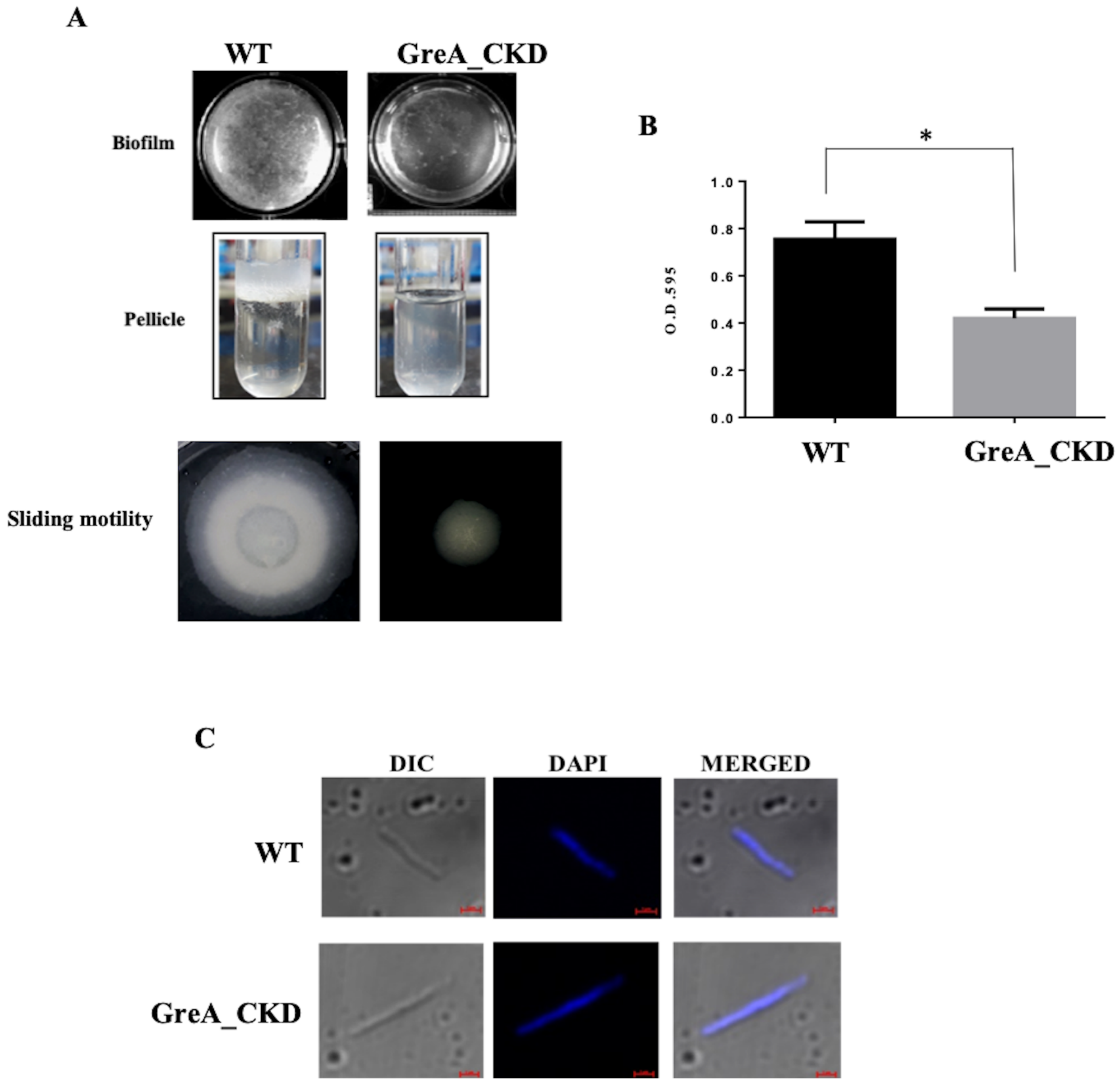

Figure S1 . Altered phenotypes of GreA_CKD. (A) Sliding motility was determined by growing the cells on 0.4% agar. Biofilm and pellicle formation were seen in static cultures without tween80. **(B)** The ability to form biofilm was quantitated by crystal violet staining. Paired t-test was used for statistical analysis. P-value < 0.05 was considered as significant, * ≤0.05 **(C)** ATc treated exponential phase cultures of WT and GreA_CKD were stained with DAPI, visualized by fluorescence microscopy under 100X lens. Experiments were carried out thrice with similar results and representative images are shown.

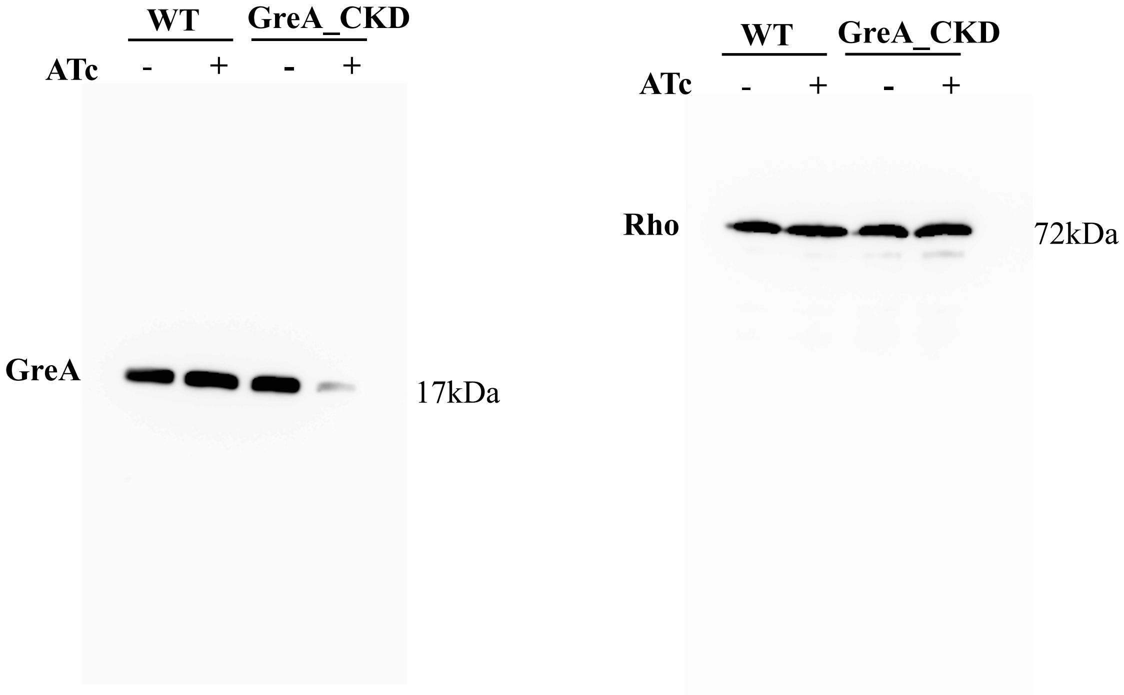

Figure S2: Full blot of the western data represented in Figure 1C.

Name	Description	References
M. smegmatis mc^2 155 (WT)	A high efficiency transformation strain of *M. smegmatis*	Laboratory stock
E. coli DH10B	A high efficiency transformation strain of *E. coli*	Laboratory stock
pRH2502	Expression of dcas9 D10A H840A from a TetR-regulated uvtetO promoter	Singh et al., Nucleic Acids Res. 44 (2016) e143
pRH2521	Expression of sgRNA from a *imyc* promoter	Singh et al., Nucleic Acids Res. 44 (2016) e143
GreA_CKD	GreA conditional knockdown of *M. smegmatis*	This study
dCas9-*M. smegmatis*	dCas9 expressing strain of *M. smegmatis*	This study

Supplementary Table S1: Strains and plasmids used in the study

A

Oligos	Forward- sequence (5'-3')	Reverse- sequence (5'-3')
SgRNA greA	GGGACTTGGGGGCCTCGCCGACCT	AAACAGGTCGGCGAGGCCCCCAAG
carD	TCTGACCGTTCGAGTACCAG	TCTCCAGGTTGGCCTTGTAG
nusB	GGTGTGGGAGTTGCTCCA	CGTCACGAGCATCACCTG
nusE	ATTCGGTCTCCGCACAAGTA	GATGTTGACGTCGACGCTTG
5S rRNA	CACAGCGGCAGGGAAACG	GTTCGGCGGTGTCCTACTT
gro L1	GCCAACATCGTGCGCGTC	CGTACTCACCGGTCGCGG
gro S	CGAGGGTGACACCGTCAT	GGCCGACAGGATCAGGTACT
dnaK	GCCGAGTCGCTGGTCTAC	CCTTCGAGCGCCTTCTTGG
nusA	ATCGACTACGACGAGGACCC	GCGAGAGCTGGAAATCGG
nusG	GTGAAGGCCAACCTCGAGA	ACCTTGCGGTTGACCTGC
sigA	AAGAGCTCGCCAAGGAGAT	TGAAGTCACCGAGCTGGCT
hupB	GCAGTTCAAGGCGGTTATC	CTTGGTCGCCGTCTTCTT
lsr2	GACCGTCACGCTTGTCGA	CCCACTGCTTCAGATCGTTG
gyrA	ACCAGTCCAGTGACCGTGTG	ATCCACAGCATGTTGGCG
gyrB	GAAGATCTGCAACGAGCAG	ACCAACTCACGCGCCCTTAG
topo1	CGCAAGCTGCGGTTCTC	TCGGTACGCATGTAGGTGATG
16S rRNA	ACGCGAAGAACCTTACCTGG	CCCAACATCTCACGACACGA
23S rRNA	CGTGCGCTTACAATCCGTCAGA	TGTGTGGATACGCCCTATTCAG
rpoA	ACCTGCTGGACATCCGTAAC	CTGGTGCAGCTTGATCTTCAC
rpoB	CGGTGACGGTTGGCTACAT	AGCGCTGACCACCGAACT
rpoC	AAGCCGGAGACCATCAACTAC	CGCTTGTACTTGCCGCAGTA
rpoZ	CCGCCAGATCCAACGATTACTAC	GGCCGACGTATTCGAGGAT
greA	AGCTGCAGGAGCTGCTCA	ATCAGGAACGTCTCGGTGTC
rho	CGCTGTACCCGAACGAGC	GGATCGTGGTCTTACCGGC

B

Northern probe	Sequence (5'-3')
16S rRNA	TTCTTCTGCACATACCGTCA
23S rRNA	TGTGTGGATACGCCCTATTCAG
5S rRNA	GTAGTATCATCGGCGCTGGCAG

Supplementary Table S2: (A) Oligonucleotides used in the study. **(B)** Probe used for northern blot assay.
